# Lack of serological and molecular evidence of arbovirus infections in bats from Brazil

**DOI:** 10.1371/journal.pone.0207010

**Published:** 2018-11-07

**Authors:** Cíntia Bittar, Rafael R. G. Machado, Manuela T. Comelis, Larissa M. Bueno, Eliana Morielle-Versute, Matheus R. Beguelini, Renato P. de Souza, Maurício L. Nogueira, Paula Rahal

**Affiliations:** 1 Laboratório de Estudos Genômicos, Instituto de Biociências, Letras e Ciências Exatas, UNESP—Universidade Estadual Paulista “Júlio de Mesquita Filho”, São José do Rio Preto, São Paulo, Brazil; 2 Laboratório de Chiroptera, Instituto de Biociências, Letras e Ciências Exatas, Universidade Estadual Paulista “Júlio de Mesquita Filho”, São José do Rio Preto, São Paulo, Brazil; 3 Centro das Ciências Biológicas e da Saúde, UFOB—Universidade Federal do Oeste da Bahia, Barreiras, Bahia, Brazil; 4 Núcleo de Doenças de Transmissão Vetorial, Instituto Adolfo Lutz, São Paulo, Brazil; 5 Laboratório de Pesquisa em Virologia, FAMERP-Faculdade de Medicina de São José do Rio Preto, São José do Rio Preto, São Paulo, Brazil; Keele University Faculty of Natural Sciences, UNITED KINGDOM

## Abstract

Viruses are important agents of emerging zoonoses and are a substantial public health issue. Among emerging viruses, an important group are arboviruses, which are characterized by being maintained in nature in cycles involving hematophagous arthropod vectors and a wide range of vertebrate hosts. Recently, bats have received increasing attention as an important source for the emergence of zoonoses and as possible viral reservoirs. Among the arboviruses, there are many representatives of the genera *Flavivirus* and *Alphavirus*, which are responsible for important epidemics such as Dengue virus, Zika virus and Chikungunya virus. Due to the importance of analyzing potential viral reservoirs for zoonosis control and expanding our knowledge of bat viruses, this study aimed to investigate the presence of viruses of the *Alphavirus* and *Flavivirus* genera in bats. We analyzed serum, liver, lungs and intestine from 103 bats sampled in northeast and southern Brazil via Nested-PCR and the hemagglutination inhibition test. All samples tested in this study were negative for arboviruses, suggesting that no active or past infection was present in the captured bats. These data indicate that the bats examined herein probably do not constitute a reservoir for these viruses in the studied areas. Further studies are needed to clarify the role of bats as reservoirs and sources of infection of these viral zoonoses.

## Introduction

Among emerging viruses, an important group are arboviruses (arthropod-borne viruses), which are characterized by being maintained in nature in cycles involving hematophagous arthropod vectors and a wide range of hosts [1,2]. These hosts are often vertebrates, especially mammals and birds.

Bats are mammals belonging to the order Chiroptera [3], and they are considered to be one of the most abundant, diverse and geographically distributed vertebrates in the world [4]. In Brazil, there is a great diversity of bats, with approximately 179 species (10 of which are endemic) and 68 subspecies belonging to 68 genera documented [5,6]. They present a broad geographic distribution, being able to fly long distances and often coming into direct or indirect contact with humans [3]. Recently, these vertebrates have received increasing attention as an important source for the emergence of zoonoses and possibly as viral reservoirs [7–9]. The infection of bats by arboviruses has long been reported by several authors [8,10–14]. Although the possibility of them acting as reservoirs has been raised, it is not clear yet if they play this role in the ecological cycle [15,16].

Being a large highly populated tropical country with one third of its territory covered by forests, Brazil presents ideal conditions for the existence of many arboviruses [17]. More than 200 arboviruses have been isolated in the country, and approximately 40 of these viruses cause diseases in humans [17,18]. The country presents a constant risk of emergence and re-emergence of arboviruses due to the existence of densely populated cities infested by mosquitoes of the genera *Culex* and *Aedes*, which are important vectors for arboviruses [1,7,19,20].

Focusing on the importance of the analysis of potential sources of zoonoses and viral reservoirs for the control of emerging viruses, the aim of this study was to investigate arbovirus infections, of the *Alphavirus* and *Flavivirus* genera, in bats from southeast and northeast Brazil. In addition, we aim to provide relevant information that may contribute to the epidemiological surveillance of diseases of great public health impact.

## Materials and methods

### Ethics statement

The animals were collected under environmental licenses from the Brazilian Institute of Environment and Natural Renewable Resources (IBAMA), SISBIO n°: 21707–1 (Process: 02027.001957 / 2006–02); 46190–1 and handled according to the Guide for the Care and Use of Laboratory Animals of the Institute for Laboratory Animal Resources Commission on Life Sciences National Research Council [21]. In addition, it should be noted that this project was approved by the Ethics Committee on the Use of Animals of the Institute of Biosciences, Letters and Exact Sciences (CEUA / IBILCE—Protocol 135/2016).

### Study areas

Sampling was performed between 2014 and 2017 at ten different sites in urban and peri-urban areas of two cities from two different states of Brazil: São José do Rio Preto, state of São Paulo (SP) and Barreiras, state of Bahia (BA). The complete geographical coordinates address and ecological characteristics of all sampling sites are available as supplementary information (S1 Table). Additionally, climatological characteristics (temperature and rainfall) are given in S1 Fig (“A” for São José do Rio Preto—SP and “B” for Barreiras-BA).

### Samples

Bats were collected using mist nets, and the sex and species of the animals were determined [22–24]. Following euthanasia, which was performed by subcutaneous anesthesia with 80 mg/kg ketamine (Dopalen-Vertebrands, Paulínia, SP, Brazil) and 20 mg/kg xylazine (Rompun-Bayer S.A., São Paulo, SP, Brazil), the liver, intestines and lungs from each specimen were removed and stored at -150°C. Blood was also collected, and serum was separated and stored at -150°C. Finally, the animals were fixed in 10% formaldehyde for 24 hours and deposited in 70% alcohol in the Chiroptera Collection of the Department of Zoology and Botany—IBILCE / UNESP, where they are available for taxonomic studies.

### RNA extraction and cDNA synthesis

The RNA of the lungs, intestines and liver was extracted in order to test for the presence of *Alphavirus* and *Flavivirus* RNA. Tissues were homogenized in a Turrax-MA102 (Marconi, Piracicaba, SP, Brazil), and pellets were used for RNA extractions in TRIzol (Thermo Fisher Scientific, Waltham, MA, USA), according to manufacturer’s instructions. Finally, the RNA was resuspended in 100 μl of water treated with DEPC (Sigma Aldrich, St. Louis, Missouri, USA) and stored at -150°C. Quantification of samples was performed on the NanoDrop 2000 spectrophotometer (Thermo Fisher Scientific, Waltham, MA, USA). Nucleic acids extracted from the organs of individual bats were subjected to cDNA synthesis using the High-Capacity cDNA Reverse Transcription *Kit* (Applied Biosystems, Foster City, CA, USA).

### Endogenous control amplification

After cDNA synthesis, in order to check the RNA quality, PCR was performed for the endogenous control, β-actin, as described previously [25].

### Molecular detection of *Alphavirus* and *Flavivirus*

Detection of viral RNA in the tissue samples was carried out by Nested-PCR assay. We designed a set of primers targeting the nsp4 region of *Alphavirus* based on 23 viruses from this genus (S1 File). To test for *Flavivirus*, we used PCR primers external to *Flav100F* and *Flav200R*, published by Maher-Sturgess et al., 2008 [26], for the Nested-PCR reaction, which targets the NS5 region. The sets of primers used for each reaction is described in Table 1. Additional information on primer design and molecular tests are available in S1 and S2 Files. The PCR reaction was carried out using Long PCR Enzyme Mix (Thermo Fisher Scientific, Waltham, MA, USA). Amplification conditions were 3 min at 94°C, 35 cycles at 94°C for 1 min, annealing at 50°C (for *Alphavirus*) and 48°C (for *Flavivirus*) for 45 seconds, and extension at 72°C for 1 min followed by a final extension at 72°C for 10 minutes. The Nested-PCR reaction was performed as described above except for the annealing temperature, 45°C (for *Alphavirus*) and 42°C (for *Flavivirus*). Samples were resolved in 1% agarose gels. The expected sizes of the amplicons were 803 bp for *Flavivirus* and 913 bp for *Alphavirus*.

**Table 1:** Sequences 5’-3’ of *Alphavirus* and *Flavivirus* specific primers used for Nested-PCR reactions.

	PCR reaction		Nested-PCR reaction	
	Name	Sequence 5’-3’	Name	Sequence 5’-3’
***Alphavirus***	α.nsP4.PF	TAYYTDGAYATGGTIGABGG	α.nsP4.NF	TGYAAYGTIACICARATGMG
	α.nsP4.PR	GGYTTICCIADYTTVAAIAG	α.nsP4.NR	AAICCICCRCARAARTAIGG
***Flavivirus***	F.NS5P-F	ACIMTBITSTGTGACATHGGIGA	Flav100F*	AAYTCIACICAIGARATGTAY
	F.NS5P-R	GTBAHIGTGTTIARGGCRTAIGT	Flav200R*	CCIARCCACATRWACCA

*Flav100F and Flav200R primers were published by Maher-Sturgess et al., 2008 [26].

### *Alphavirus* and *Flavivirus* antibody detection

The presence of antibodies for *Alphavirus* and *Flavivirus* in the serum samples were tested by hemagglutination inhibition (HI), as described previously [27]. Additional information about the antigens and the positive controls used in the HI, are given in S2 Table. The tests were performed by the Adolfo Lutz Institute–SP.

## Results

**Fig 1:**
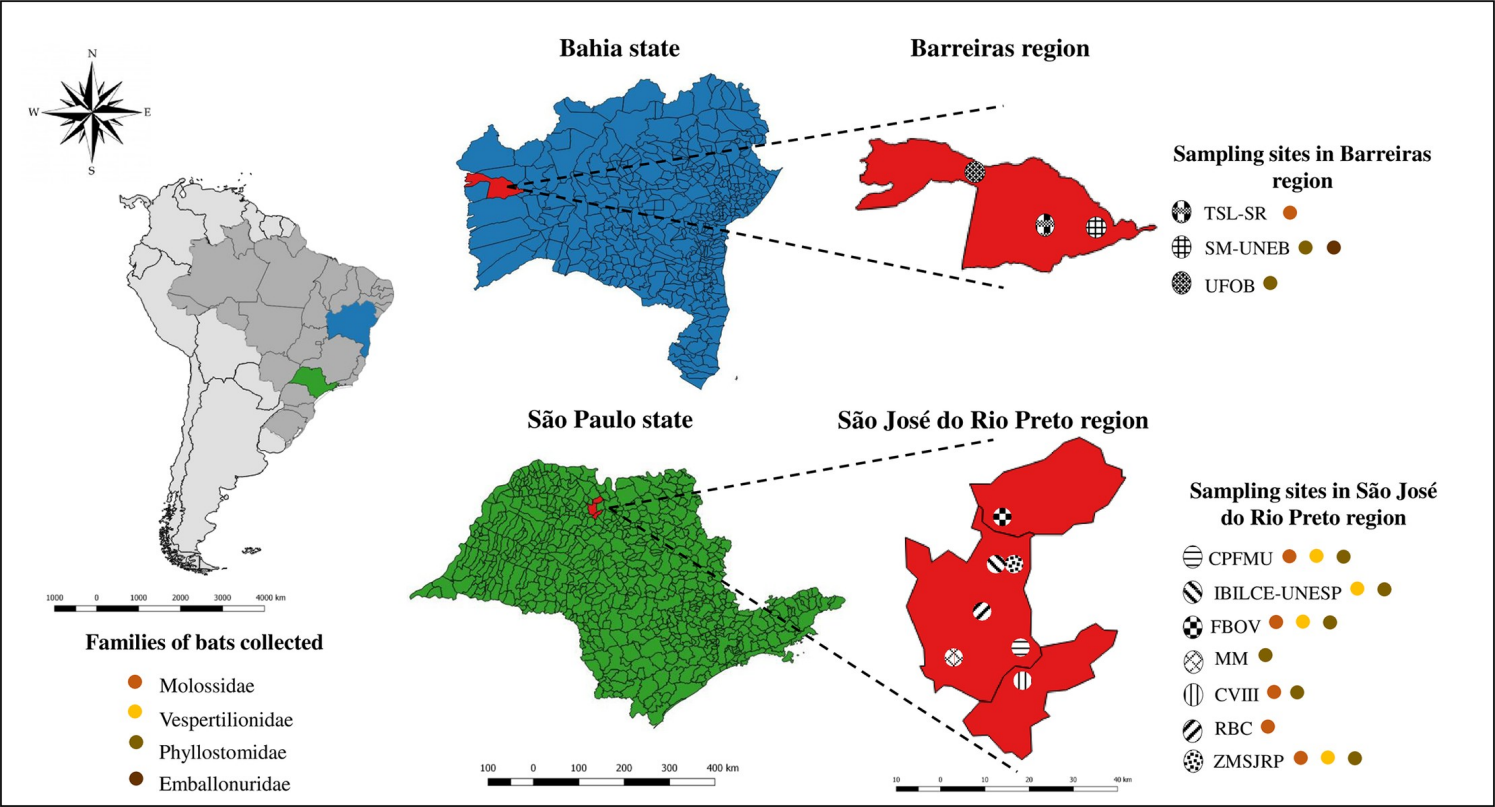
Locations of bat collections and the families found in each sampling site. On the left is the map of Latin America, highlighting the two Brazilian states, Bahia (in blue) and São Paulo (in green) in which bats were collected. At greater magnification, the collection sites within the regions of São José do Rio Preto–SP and Barreiras–BA are shown. The families of bats collected are represented by colored circles. The image was designed using QGIS 2.18.15 software (http://www.qgis.org/en/site/about/index.html).

A total of 39 animals were collected in Barreiras-BA and 64 in the region of São José do Rio Preto-SP, totaling 103 bats. These were distributed in four families: Molossidae, Phyllostomidae, Emballonuridae and Vespertilionidae (Fig 1 and Table 2). The diversity of species and the amount of male/female per species are reported in Table 2. The livers and intestines were collected from 28 specimens from Barreiras–BA and only the livers from 11 specimens. From the region of São José do Rio Preto-SP liver, lungs and intestines were collected from all bats. Detailed information on species, sex, organs tested, site of sampling and month of collection for each specimen are presented in S3 Table. The necropsies did not show any morphological changes that indicated a pathological condition.

**Table 2:** Diversity of the species collected in relation to the sex of each specimen.

Family	Species	Number of specimens	
		Male	Female
Molossidae	*Eumops glaucinus*	-	1
	*Molossops temminckii*	1	-
	*Molossus molossus*	6	18
	*Molossus rufus*	14	4
Vespertilionidae	*Eptesicus furinalis*	1	2
	*Eptesicus sp*	-	1
	*Myotis nigricans*	1	-
Phyllostomidae	*Artibeus lituratus*	17	5
	*Artibeus planirostris*	9	3
	*Carollia perspicillata*	1	-
	*Desmodus rotundus*	1	-
	*Diphylla ecaudata*	2	-
	*Glossophaga soricina*	2	1
	*Phyllostomus discolor*	3	2
	*Phyllostomus hastatus*	1	-
	*Platyrrhinus lineatus*	1	-
	*Sturnira lilium*	1	-
	*Vampyressa pussilla*	-	1
Emballonuridae	*Peropteryx leucoptera*	1	-

RNA from all tissue samples was extracted and quantified. The quality of the RNA was confirmed by the amplification of the endogenous gene, β-actin, from all tested samples.

The presence of *Alphavirus* and *Flavivirus* RNA was investigated by Nested-PCR in all available tissue samples. The results revealed that none of the 103 samples were infected by *Alphavirus* and *Flavivirus* at the time they were collected.

We also tested for serological evidence of previous infections by viruses from this groups using HI. A total of 73 serum samples (46 from São José do Rio Preto–SP and 27 from Barreiras–BA) were tested for anti-DENV-1, anti-DENV-2, anti-DENV-3, anti-DENV-4, anti-MAYV, anti-WEEV, anti-EEEV, anti-YFV, anti-SLEV, anti-ROCV, anti-ILHV, anti-MUCV and anti-IGUV antibodies. No HI was detected in any of the bats’ serum or in the negative serum used as a control, indicating no previous infection by any of these viruses.

## Discussion

The role of bats in the transmission and ecology of arboviruses, such as *Flavivirus* and *Alphavirus*, is not fully elucidated; however, several studies over the past 40 years have demonstrated that bats are susceptible to infection by viruses of these genera. For example, evidence of nucleic acids and antibodies from the four DENV serotypes has been reported in bats in Central and South America [28]. In addition, CHIKV was detected in bats from Asia [29]. In this study, we analyzed the presence of RNA from *Alphavirus* and *Flavivirus* and antibodies from viruses from these genera in animals collected in São José do Rio Preto (São Paulo State) and Barreiras (Bahia State). The first city is in the northwest region of São Paulo state and comprises an area with a high incidence of arbovirus infection in humans. Several studies have shown that this is a region of YFV transmission [30,31], is hyper-endemic for DENV [32–34], has confirmed cases of MAYV [35,36], Saint Louis encephalitis virus (SLEV) [34,37] and ZIKV [38–40]. Additionally, some cases of DENV intra-serotype co-infection [32] and co-infection with other *Flaviviruses* [34,41] have been reported. In the Barreiras region, epidemiological reports from 2014 to 2017 also show the occurrence of DENV, CHIKV, ZIKV and YFV [42–44]. In addition, all animals were collected in densely populated urban or peri-urban areas where the main vector of these arboviruses, *Aedes aegypti*, has extensive circulation. Our results suggest that no active or past infections by the arboviruses in this study were present in the captured bats. These facts indicate that although the bats collected are in close contact with these viruses, that they are not being infected and that they probably do not constitute a reservoir in the studied regions.

Our study sampled bats from the Molossidae, Vespertilionidae, Phyllostomidae and Emballonuridae families. The few previous studies that sought to identify arboviruses in these bat families show that Molossidae and Phyllostomidae have the highest incidence of arbovirus infection. As an example of Molossidae infection, St. Louis encephalitis virus (SLEV) was isolated from *Tadarida brasiliensis mexicana* in Texas [45]. Additionally, in east Africa, antibodies reactive to ZIKV were detected at a high seroprevalence in *Mops condylurus* using the indirect hemagglutination test (HAI) [10]. In the Phyllostomidae family, there is a single work that detected the viral RNA of DENV-1 in *Carollia perspicillata* in French Guiana [46]. Additionally, specific antibodies against West Nile Virus (WNV), SLEV and DENV 1–4 were detected by the plaque reduction neutralization test (PRNT) in three bat species from Mexico: *Glossophaga soricina*, *Artibeus jamaicensis* and *Artibeus lituratus*, with a *Flavivirus* antibody prevalence of 33%, 24%, and 9%, respectively [47]. For the Emballonuridae and Vespertilionidae families, there are no records of viral RNA or antibodies against the *Flavivirus* and *Alphavirus* genera [46].

Even with the absence of studies that reported the lack of active and/or past arbovirus infection in bats, some studies with DENV corroborate the results obtained in this work. In a study carried out in Mexico, which investigated the presence of the 4 serotypes of DENV in serum, lung and liver of 240 bats, no active or past infection in these animals was found [48]. Additionally, other studies with DENV, demonstrate that some serotypes of this virus did not replicate efficiently in cell lines derived from neotropical bat species and indicate that some species are incapable of sustaining Dengue virus replication and are unlikely to act as reservoirs for this virus [49–51]. Moreover, a recent work showed that bats sampled from households in Costa Rican urban environments do not sustain DENV amplification, since they do not support sufficient virus replication. These findings excluded them as potential hosts or reservoirs with no role in the transmission cycle and more likely are functioning as epidemiological dead-end hosts for this virus [52].

To date, few studies have aimed to identify arbovirus in bats, even with all the recognition of the importance of these animals in the emergence of zoonotic viruses. Some studies have demonstrated that ecological, behavioral and phylogenetic characteristics can influence and diversify the immunological response to viral infections in different species of bats [53,54]. For example, some evidence suggests that large colonies and higher species richness were significantly positively associated with European Bat lyssavirus 1 (EBLV-1) seroprevalence [54]. Additionally, they observed that EBLV-1 seroprevalence in bats from the Vespertilionidae and Rhinolophidae families were different. This difference is likely due to differences between bat species in the immune response and the lifespan of immunity to a virus infection [53,55]. Therefore, it is more likely to find seropositive bats in species with long lifespan immunity [55]. However, no study aimed to investigate differences in immunological responses between bat species to arbovirus infection.

Even though there is no serological and molecular evidence of arbovirus infections in Brazilian bats from the studied regions, we emphasize the importance of continuing studies in other locations in order to evaluate the importance of bats as arbovirus reservoirs and to determine if these animals are an important part of its enzootic cycle.

## Supporting information

S1 Fig Climatological characteristics of the sampling sites. **A**. Average monthly rainfall and temperature in the São José do Rio Preto region, from 2014 to 2017. The data were provided by the Integrated agrometeorological information center (CIIAGRO-Brazil: http://www.ciiagro.sp.gov.br). **B.** Average monthly rainfall and temperatures in the Barreiras region, from 2014–2015. The data were provided by the National Institute of Meteorology (INMET-Brazil: www.inmet.gov.br).
(TIF)

S1 Table Sampling sites information. Additional information, geographic coordinates and ecological characteristics of sampling sites. The ecological characteristics were obtained by the Brazilian company of agricultural research—Ministry of Agriculture, Livestock and Supply (EMBRAPA-Brazil: https://www.embrapa.br/) and by the Forestry Institute—Sao Paulo's State Government—Secretariat of Environment (IF-Brazil: http://iflorestal.sp.gov.br/).
(XLSX)

S2 Table HI Antigens and Positive Controls. Description of the antigens (strain, virus and sample) and the positive controls used in HI. The positive controls were pools of mouse hyperimmune sera for each subgroup of arboviruses (Pool A—*Alphavirus* and B—*Flavivirus*).
(XLSX)

S3 Table Characteristics of bats collected. Characteristics of bats collected (identification number—ID, family, species, sex, type of sample collected of each specimen and sampling site and month/year of collection).
(XLSX)

S1 File *Alphaviru*s primers design and molecular tests. (PDF)

S2 File *Flavivirus* primers design and molecular tests. (PDF)
