# Supplementary figures and images for: Lack of serological and molecular evidence of arbovirus infections in bats from Brazil
Source: PLoS One. 2018 Nov 7;13(11):e0207010. doi: 10.1371/journal.pone.0207010 (PMC6221338; doi:10.1371/journal.pone.0207010)

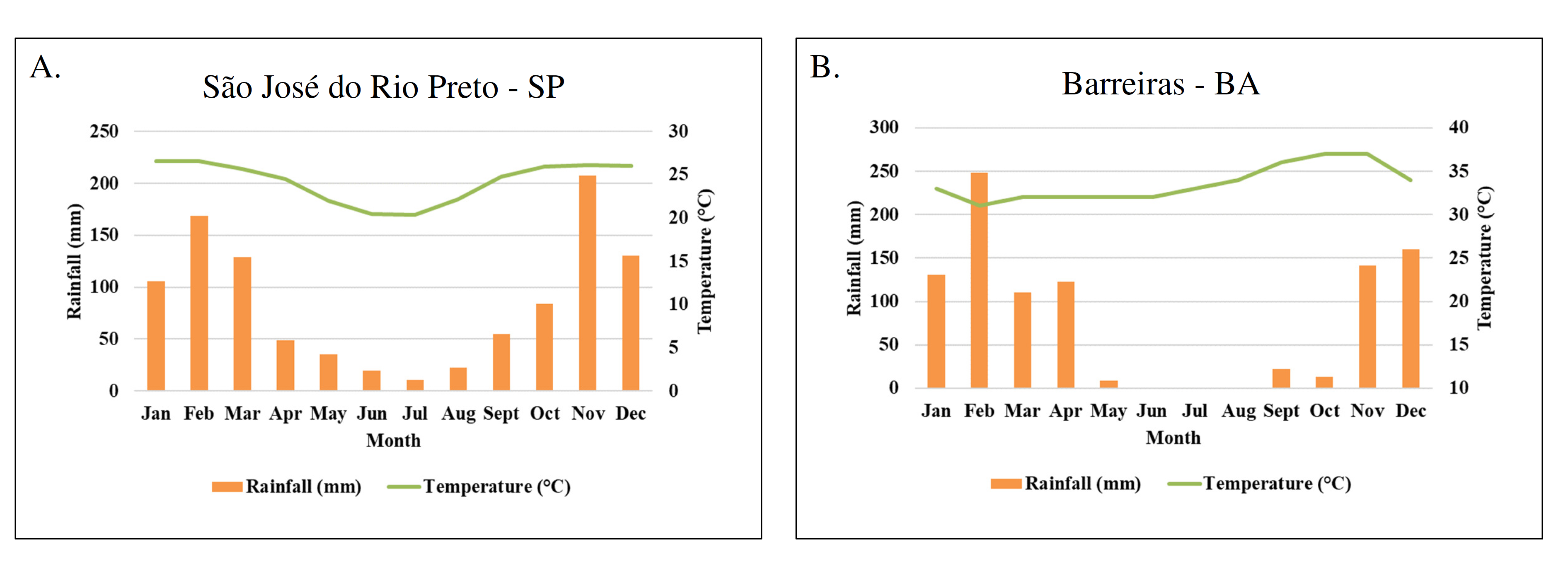

Supplement: S1 Fig — A. Average monthly rainfall and temperature in the São José do Rio Preto region, from 2014 to 2017. The data were provided by the Integrated agrometeorological information center (CIIAGRO-Brazil: http://www.ciiagro.sp.gov.br). B. Average monthly rainfall and temperatures in the Barreiras region, from 2014–2015. The data were provided by the National Institute of Meteorology (INMET-Brazil: www.inmet.gov.br). (TIF) [file pone.0207010.s001.tif]
