# Supplementary material for: Lack of serological and molecular evidence of arbovirus infections in bats from Brazil
Source: PLoS One. 2018 Nov 7;13(11):e0207010. doi: 10.1371/journal.pone.0207010 (PMC6221338; doi:10.1371/journal.pone.0207010)

Alphavirus Primers

Alignment with reference sequences

PCR – Forward (α.nsP4.PF)

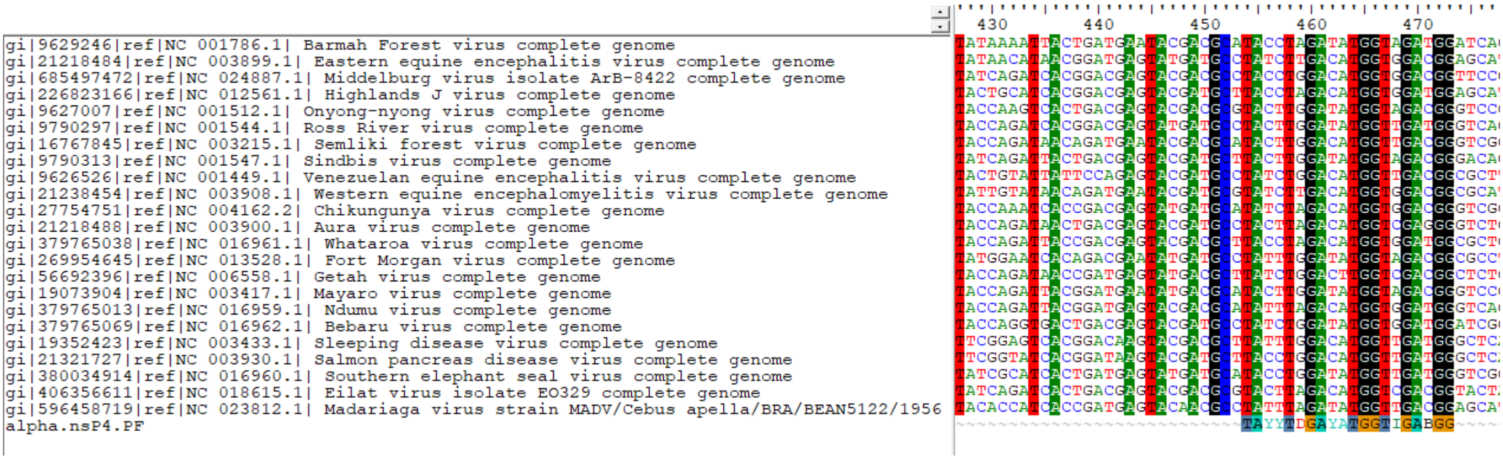

PCR – Reverse (α.nsP4.PR)

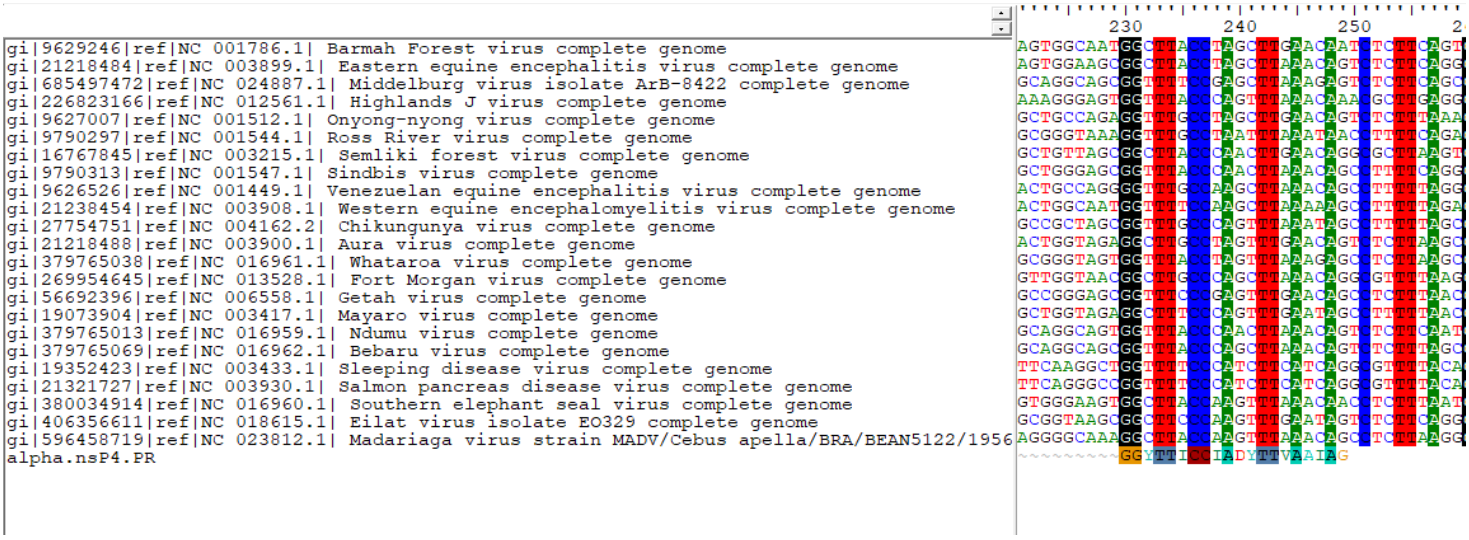

NESTED-PCR – Forward (α.nsP4.NF)

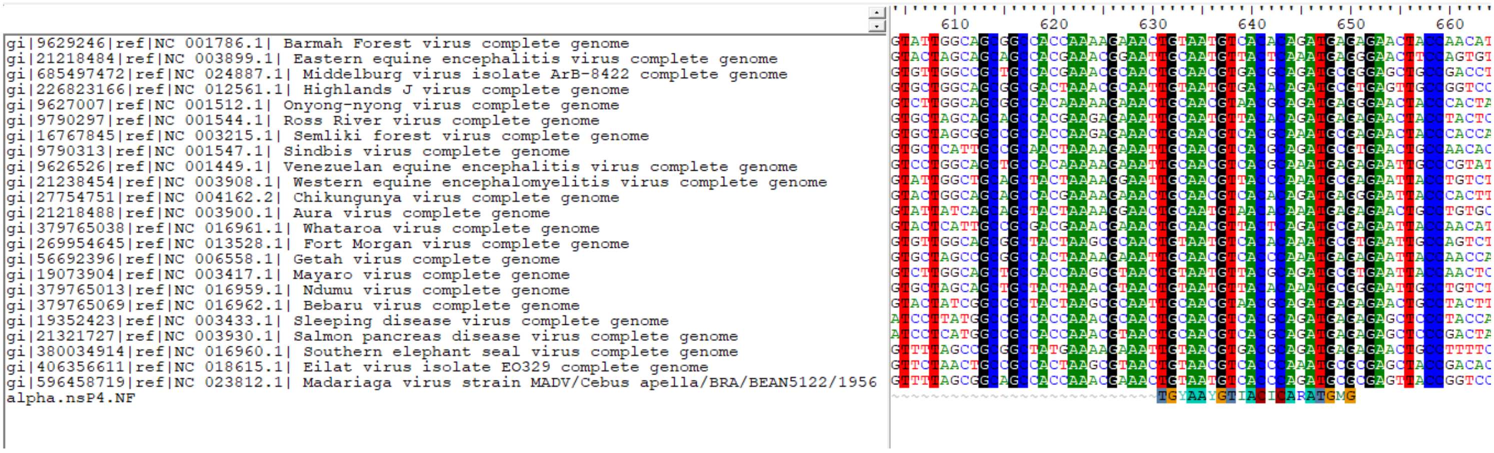

NESTED-PCR – Reverse (α.nsP4.NR)

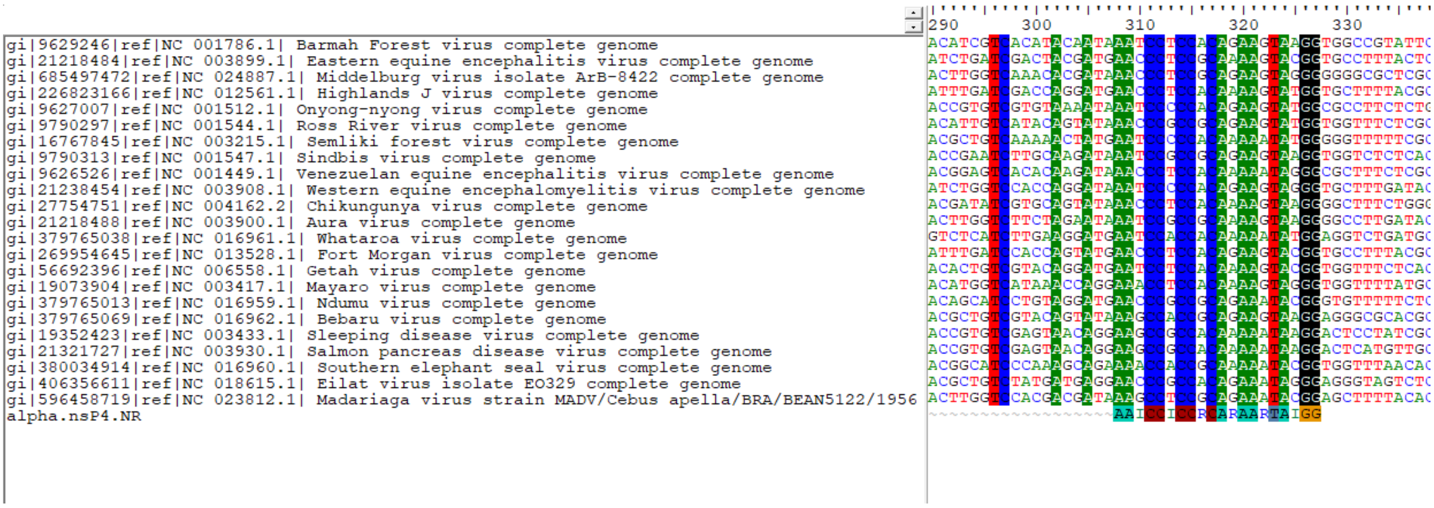

Experimetal testing of the NESTED-PCR primer set

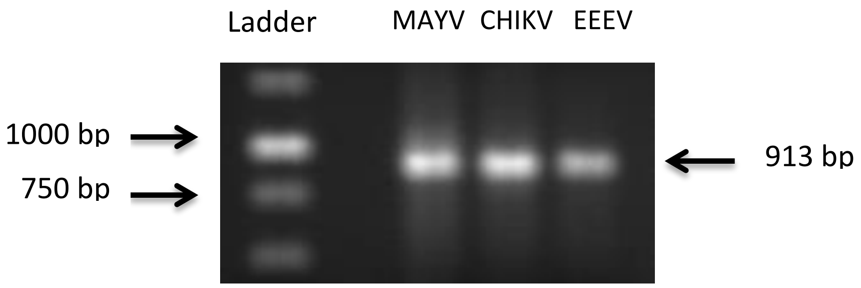

Supplement: S1 File — (PDF) [file pone.0207010.s005.pdf]
