# Supplementary material for: Lack of serological and molecular evidence of arbovirus infections in bats from Brazil
Source: PLoS One. 2018 Nov 7;13(11):e0207010. doi: 10.1371/journal.pone.0207010 (PMC6221338; doi:10.1371/journal.pone.0207010)

Flavivirus Primers

Alignment with reference sequences

PCR – Forward (F.NS5P-F)

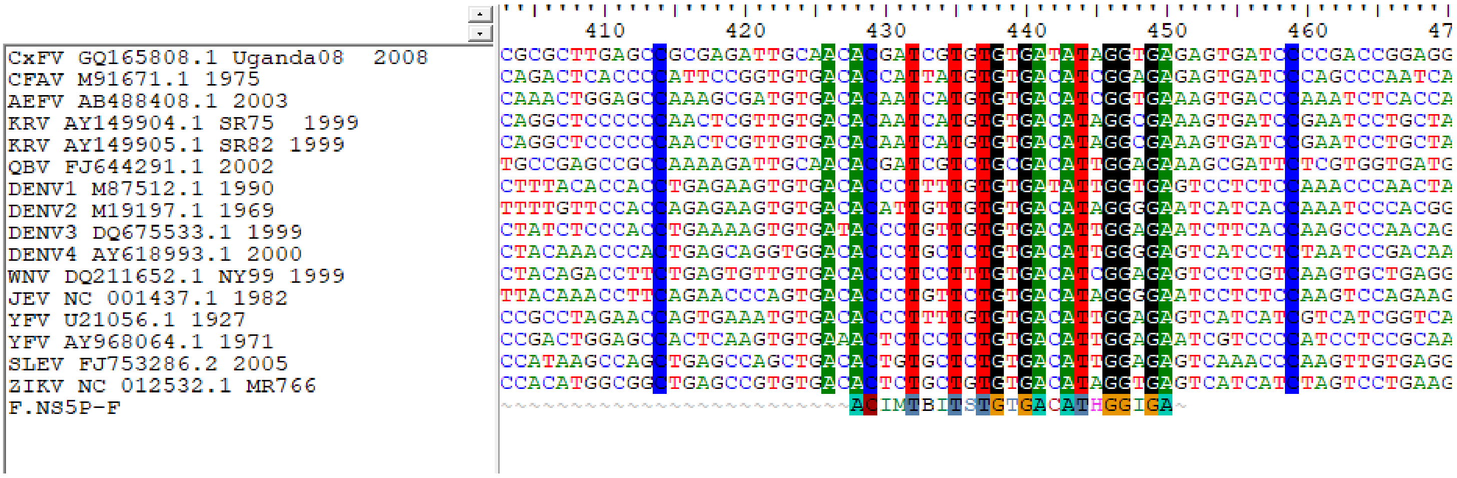

PCR – Reverse (F.NS5P-R)

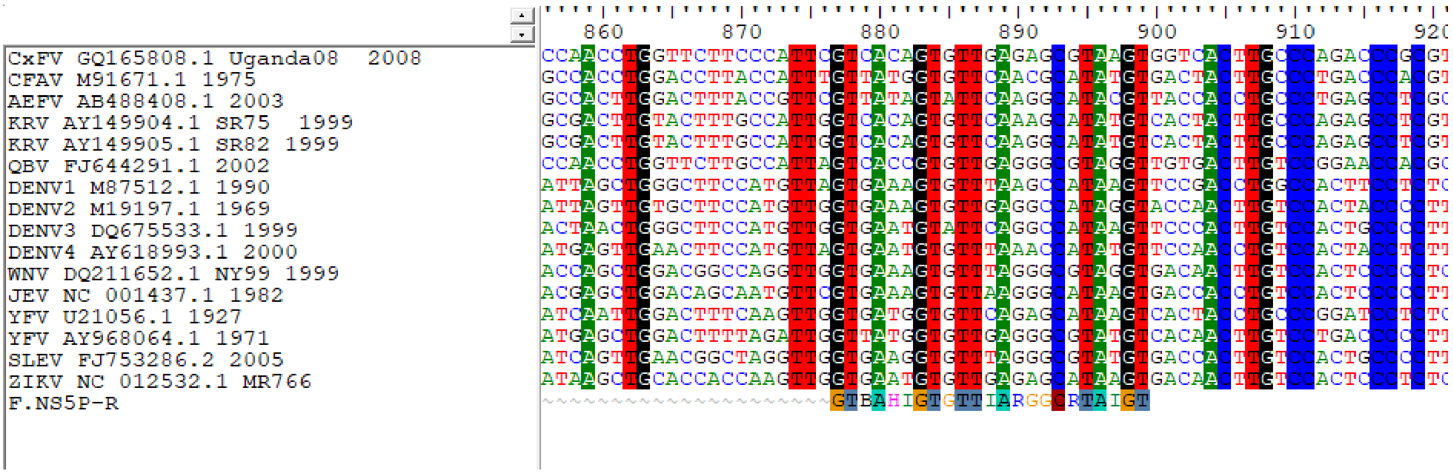

NESTED-PCR – Forward (Flav100F) *

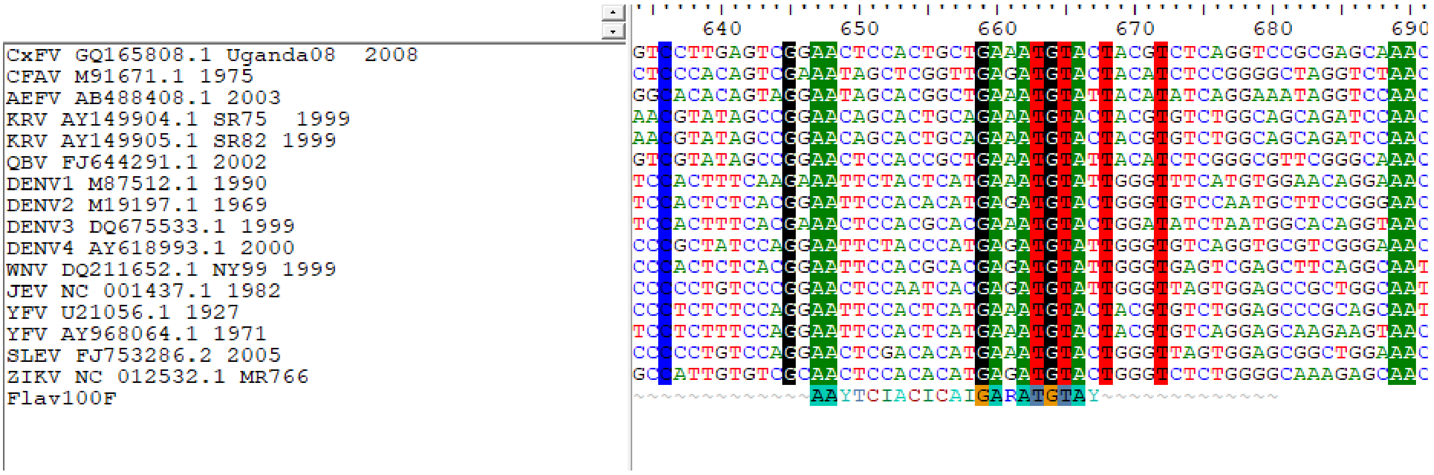

NESTED-PCR – Reverse (Flav200R)*

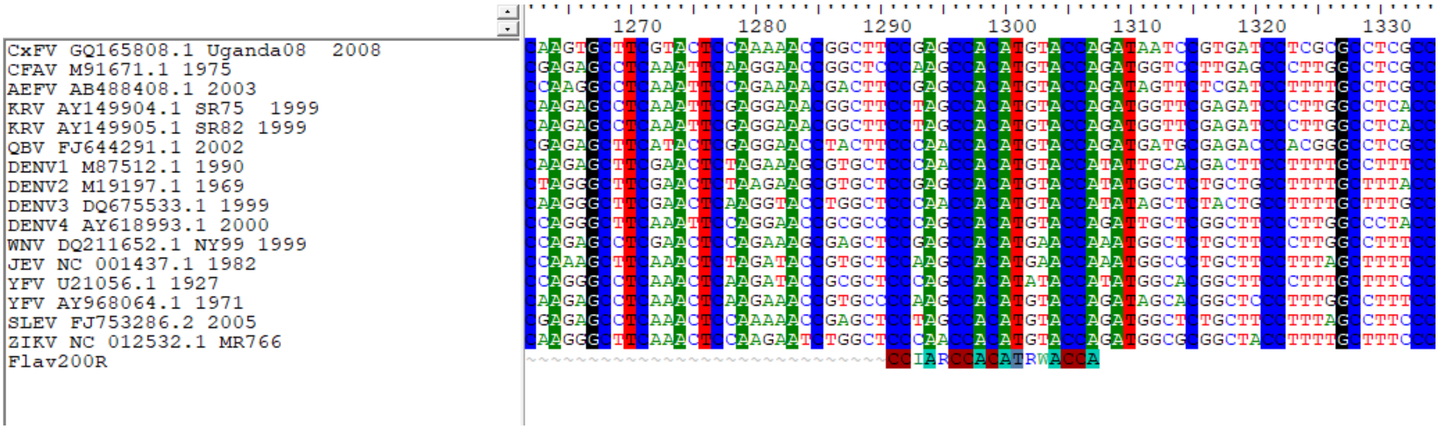

* Maher-Sturgess SL, Forrester NL, Wayper PJ, Gould EA, Hall RA, et al. (2008) Universal primers that amplify RNA from all three *Flavivirus* subgroups. Virol J 5: 16.

Experimetal testing of the NESTED-PCR primer set

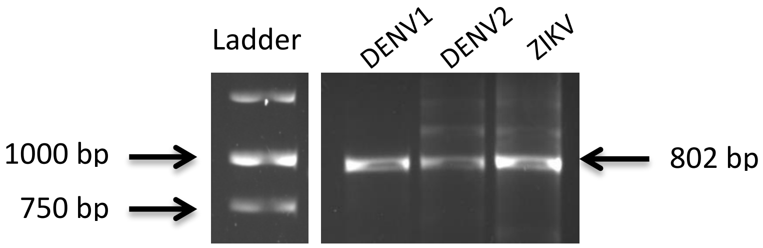

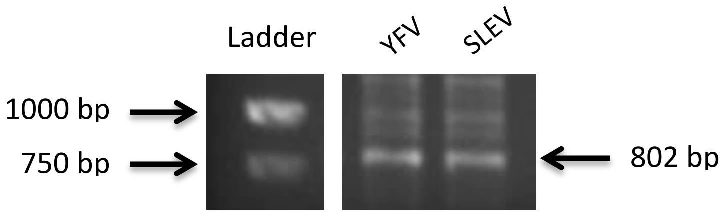

Supplement: S2 File — (PDF) [file pone.0207010.s006.pdf]
